# PREDICT underestimates survival of patients with HER2-positive early-stage breast cancer

**DOI:** 10.1038/s41523-022-00452-8

**Published:** 2022-07-20

**Authors:** Elisa Agostinetto, Lieveke Ameye, Samuel Martel, Philippe Aftimos, Noam Pondé, Christian Maurer, Sarra El-Abed, Yingbo Wang, Malou Vicente, Saranya Chumsri, Judith Bliss, Judith Kroep, Marco Colleoni, Fausto Petrelli, Lucia Del Mastro, Alvaro Moreno-Aspitia, Martine Piccart, Marianne Paesmans, Evandro de Azambuja, Matteo Lambertini

**Affiliations:** 1 Academic Trials Promoting Team, Institut Jules Bordet and l’Université Libre de Bruxelles (U.L.B), Brussels, Belgium; 2 Humanitas University, Department of Biomedical Sciences, via Rita Levi Montalcini 4, 20090 Pieve Emanuele, Milan, Italy; 3 Data Center, Institut Jules Bordet, Brussels, Belgium; 4 Department of Hemato-Oncology, CISSS Montérégie-Centre/Hôpital Charles-Le Moyne, Université de Sherbrooke, Greenfield Park, QC Canada; 5 Clinical Trials Conduct Unit, Institut Jules Bordet – Université Libre de Bruxelles, Brussels, Belgium; 6 Clinical Oncology Department, AC Camargo Cancer Center, São Paulo, Brazil; 7 University of Cologne, Department I of Internal Medicine, Center for Integrated Oncology Aachen Bonn Cologne Dusseldorf, Cologne, Germany; 8 Breast International Group (BIG), Brussels, Belgium; 9 Novartis Pharma AG, Basel, Switzerland; 10 Institut Jules Bordet, Université Libre de Bruxelles, Brussels, Belgium; 11 Robert and Monica Jacoby Center for Breast Health, Mayo Clinic, Jacksonville, FL USA; 12 The Institute of Cancer Research, Clinical Trials & Statistics Unit, London, UK; 13 Department of Medical Oncology, Leiden University Medical Center, P.O. Box 9600, 2300 RC Leiden, The Netherlands; 14 IEO European Institute of Oncology, IRCCS, Milan, Italy; 15 Oncology Unit, ASST Bergamo Ovest, Treviglio (BG), Italy; 16 Department of Medical Oncology, U.O. Clinica di Oncologia medica, IRCCS Ospedale Policlinico San Martino, Genova, Italy; 17 Department of Internal Medicine and Medical Specialties (DiMI), School of Medicine, University of Genova, Genova, Italy

**Keywords:** Breast cancer, Targeted therapies

## Abstract

The prognostic performance of PREDICT in patients with HER2-positive early breast cancer (EBC) treated in the modern era with effective chemotherapy and anti-HER2 targeted therapies is unclear. Therefore, we investigated its prognostic performance using data extracted from ALTTO, a phase III trial evaluating adjuvant lapatinib ± trastuzumab vs. trastuzumab alone in patients with HER2-positive EBC. Our analysis included 2794 patients. After a median follow-up of 6.0 years (IQR, 5.8–6.7), 182 deaths were observed. Overall, PREDICT underestimated 5-year OS by 6.7% (95% CI, 5.8–7.6): observed 5-year OS was 94.7% vs. predicted 88.0%. The underestimation was consistent across all subgroups, including those according to the type of anti HER2-therapy. The highest absolute differences were observed for patients with hormone receptor negative-disease, nodal involvement, and large tumor size (13.0%, 15.8%, and 15.3%, respectively). AUC under the ROC curve was 73.7% (95% CI 69.7–77.8) in the overall population, ranging between 61.7% and 77.7% across the analyzed subgroups. In conclusion, our analysis showed that PREDICT highly underestimated OS in HER2-positive EBC. Hence, it should be used with caution to give prognostic estimation to HER2-positive EBC patients treated in the modern era with effective chemotherapy and anti-HER2 targeted therapies.

## Introduction

The addition of trastuzumab to adjuvant chemotherapy has dramatically improved the outcomes of patients with HER2-positive early breast cancer, reducing the risk of mortality by more than 30%^1^. Despite the undoubted benefit of adjuvant therapy, several clinical questions remain open. Approximately 25% of patients still experience recurrence up to 10 years from diagnosis, and further research efforts are needed to better refine patient selection for adopting escalation or de-escalation treatment strategies^2,3^.

PREDICT (www.predict.nhs.uk) is a publicly available, online tool that helps to predict the individual prognosis of patients with early breast cancer and to show the impact of adjuvant treatments administered after breast cancer surgery. It uses traditional clinical-pathological factors, and it is aimed to support clinical decision making in the adjuvant setting. The original version of PREDICT (v.1.0) was derived from cancer registry information on 5,694 women treated in East Anglia from 1999–2003, and was subsequently validated in several datasets of patients with breast cancer^4,5^. In 2011, the model was updated to include HER2 status. Estimates for the prognostic effect of HER2 status were based on an analysis of 10,179 cases collected by the Breast Cancer Association Consortium (BCAC), none of which had been diagnosed after 2004, to ensure that patients did not receive trastuzumab^6^. A subsequent validation was done in 2012 in a British Columbia Canadian cohort^7^. This study demonstrated that the inclusion of the HER2 status allowed the model to perform better than the previous PREDICT version and Adjuvant! Online in estimating overall and breast-cancer-specific survival^7^.

Although the use of PREDICT is recommended to aid decision making in the adjuvant setting^8^, its prognostic role in HER2-positive early breast cancer patients treated with modern chemotherapy and anti-HER2 therapies remains unclear. We aimed to investigate the prognostic performance of PREDICT in patients with HER2-positive early breast cancer who received trastuzumab-based therapy started concurrently with chemotherapy within the ALTTO trial. The ALTTO trial is the largest adjuvant study ever conducted in the field of HER2-positive early breast cancer and, including at least 5-year follow-up data from all patients^9^, represented a unique opportunity to investigate the reliability and prognostic performance of PREDICT in women with HER2-positive disease.

## Results

**Fig. 1:**
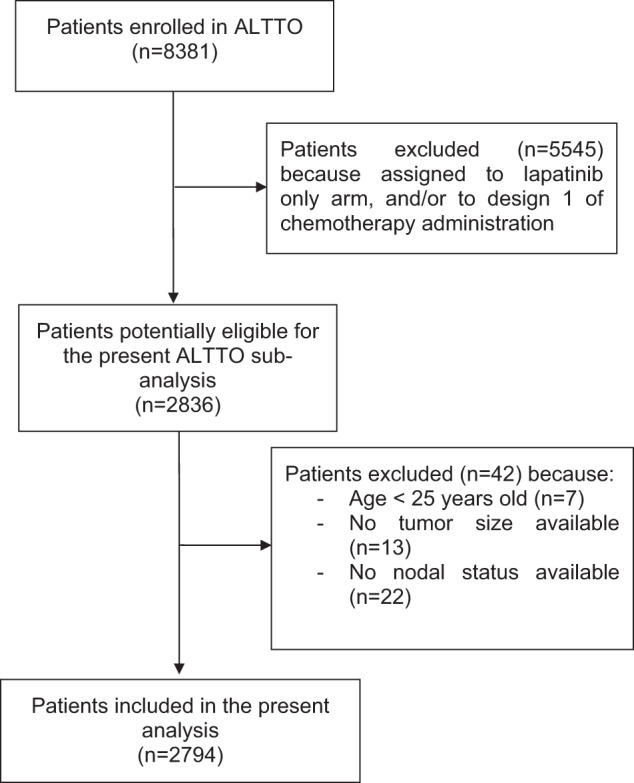
STROBE flow-chart. This figure illustrates the patient selection process.

Out of 8381 patients included in the ALTTO trial, 2836 were treated with chemotherapy and concurrent trastuzumab-based therapy and were potentially eligible for the present analysis. In 42 patients, the PREDICT algorithm was not evaluable (due to age of the patient <25 years old [*n* = 7], missing tumor size [*n* = 13], or missing lymph nodes status [*n* = 22]). Therefore, 2794 patients were included in the present analysis (Fig. 1).

Most patients (71%) were aged between 41 and 64 years (Table 1). Twenty-five percent of patients had negative nodal status, 45% had a tumor size ≤2 cm and 58% had hormone receptor-positive disease. Regarding administered treatments, 88% underwent an anthracycline-based chemotherapy regimen (design 2). The majority of patients with hormone receptor-positive disease (45%) received a selective estrogen receptor modulator (SERM) (tamoxifen).

**Table 1:** Characteristics of the patients (overall and per randomization arm).

	All patients	Trastuzumab + lapatinib	Trastuzumab alone	Trastuzumab followed by lapatinib
**N (%)**	2794 (100.0)	925 (100.0)	936 (100.0)	933 (100.0)
*Age at randomization*				
≤40	495 (17.7)	161 (17.4)	160 (17.1)	174 (18.7)
41–64	1989 (71.2)	667 (72.1)	667 (71.3)	655 (70.2)
≥65	310 (11.1)	97 (10.5)	109 (11.7)	104 (11.2)
*Ethnicity*				
Asian	606 (21.7)	201 (21.7)	200 (21.4)	205 (22.0)
Black	59 (2.1)	28 (3.0)	14 (1.5)	17 (1.8)
White	2001 (71.6)	657 (71.0)	677 (72.3)	667 (71.5)
Other/missing	128 (4.6)	39 (4.2)	45 (4.8)	44 (4.7)
*Histology*				
Ductal	2605 (93.2)	868 (93.8)	867 (92.6)	870 (93.2)
Lobular	101 (3.6)	34 (3.7)	38 (4.1)	29 (3.1)
Others	119 (4.3)	35 (3.8)	43 (4.6)	41 (4.4)
*Central HR status*				
Negative	1185 (42.4)	393 (42.5)	398 (42.5)	394 (42.2)
Positive	1609 (57.6)	532 (57.5)	538 (57.5)	539 (57.8)
*Number of positive lymph nodes*				
0	567 (25.5)	180 (24.7)	200 (26.5)	187 (25.3)
1–3	945 (42.6)	319 (43.8)	314 (41.6)	312 (42.3)
≥4	709 (31.9)	230 (31.6)	240 (31.8)	239 (32.4)
*Tumor size (mm)*				
≤20 mm	1248 (44.7)	397 (42.9)	436 (46.6)	415 (44.5)
21–50 mm	1356 (48.5)	466 (50.4)	439 (46.9)	451 (48.3)
>50 mm	190 (6.8)	62 (6.7)	61 (6.5)	67 (7.2)
*Tumor grade*				
1	79 (2.8)	26 (2.8)	22 (2.4)	31 (3.3)
2	936 (33.6)	310 (33.6)	295 (31.6)	331 (35.6)
3	1698 (60.9)	561 (60.9)	589 (63.0)	548 (58.9)
X (differentiation cannot be assessed)	75 (2.7)	25 (2.7)	29 (3.1)	21 (2.3)
*Surgery*				
BCS	1226 (43.9)	399 (43.1)	408 (43.6)	419 (44.9)
Mastecomy	1538 (56.1)	526 (56.9)	529 (56.4)	514 (55.1)
*Type of CT*				
Non-anthracycline based	322 (11.5)	103 (11.1)	109 (11.7)	110 (11.8)
Anthracycline-based	2472 (88.5)	822 (88.9)	827 (88.4)	823 (88.2)
*Type of endocrine therapy*				
AI	581 (39.4)	192 (39.4)	197 (40.3)	192 (38.5)
AI & SERM	212 (14.4)	68 (14.0)	77 (15.8)	67 (13.4)
LHRH	11 (0.8)	4 (0.8)	5 (1.0)	2 (0.4)
SERM	671 (45.5)	223 (45.8)	210 (42.9)	238 (47.7)

*HR* hormone receptors, *BCS* breast conserving surgery, *CT* chemotherapy, *AI* aromatase inhibitors, *SERM* selective estrogen receptor modulators, *LHRH* luteinizing hormone-releasing hormone.

Median follow-up of included patients was 6.0 years (interquartile range: 5.8–6.7). Overall, 182 deaths were observed.

### Calibration

**Fig. 2:**
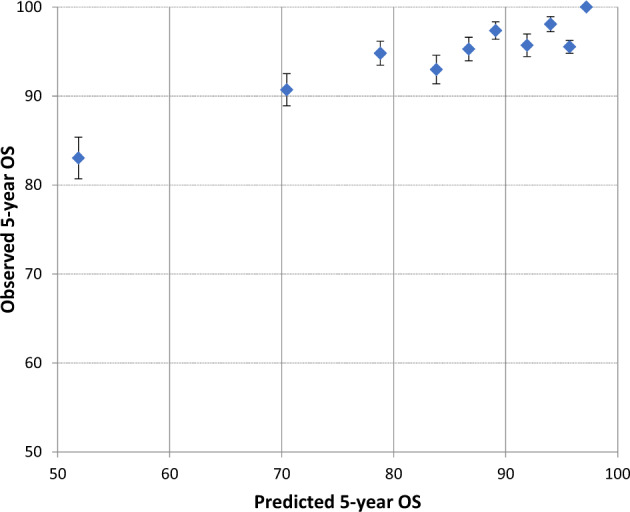
Calibration plot showing observed versus predicted 5-year overall survival: for each decile of the predicted 5-year overall survival, the mean observed 5-year overall survival is presented, with error bars presenting the standard error. OS overall survival.

Median predicted and observed 5-year overall survival (OS) were 88.0% and 94.7%, respectively (standard error 0.0044, difference −6.7%, 95% Confidence Intervals [CI] −7.5 to −5.8), thus indicating an underestimation of OS by PREDICT score (Table 2, Fig. 2).

**Table 2:** Median predicted probability of 5-year overall survival and observed 5-year overall survival rate in the study population.

		% 5 years OS			
		Predicted	Observed	s. e.	Difference (95% CI)
All patients	2794	88.00	94.69	0.44	−6.69 (−7.55 to–5.83)
*Type of anti-HER2 therapy*					
Lapatinib + trastuzumab	925	87.90	94.88	0.75	−6.98 (−8.45–5.51)
Trastuzumab alone	936	87.90	94.18	0.79	−6.28 (−7.83–4.73)
Trastuzumab followed by lapatinib	933	88.20	95.02	0.73	−6.82 (−8.25–5.39)
*Type of chemotherapy*					
Non-anthracycline-based	322	88.15	96.22	1.12	−8.07 (−10.27–5.87)
Anthracycline-based	2472	87.95	94.51	0.47	−6.56 (−7.48–5.64)
*Age at randomization*					
≤40	495	90.40	95.64	0.95	−5.24 (−7.10–3.38)
41–64	1989	88.20	94.91	0.51	−6.71 (−7.71–5.71)
≥65	310	82.05	91.78	1.61	−9.73 (−12.89–6.57)
*Central HR status*					
Negative	1185	80.20	93.15	0.76	−12.95 (−14.44–11.46)
Positive	1609	93.10	95.82	0.52	−2.72 (−3.74–1.70)
*Number of positive lymph nodes*					
0	567	91.80	97.93	0.62	−6.13 (−7.35–4.91)
1–3	945	87.40	96.40	0.63	−9.00 (−10.23–7.77)
≥4	709	71.80	87.61	1.27	−15.81 (−18.30–13.32)
*Tumor size (mm)*					
≤20 mm	1248	91.25	97.43	0.46	−6.18 (−7.08–5.28)
21–50 mm	1356	86.00	93.33	0.70	−7.33 (−8.70–5.96)
>50 mm	190	71.05	86.37	2.59	−15.32 (−20.40–10.24)

*OS* overall survival, *s.e.* standard error, *CI* confidence interval, *CT* chemotherapy, *HR* hormone receptors.

This finding was consistent across all subgroups, with a difference ranging from 2.7% (in the hormone receptor-positive subgroup) to 15.8% (in patients with ≥4 positive lymph nodes) (Table 2). The underestimation of survival by PREDICT was consistent and similar in all analyzed subgroups, including among patients treated with lapatinib and trastuzumab (Predicted—observed 5-year OS: −6.98), trastuzumab alone (Predicted – observed 5-year OS: −6.28), or trastuzumab followed by lapatinib (Predicted—observed 5-year OS: −6.82).

The highest absolute differences were observed for patients with hormone receptor-negative disease (13.0%), larger tumor size (>50 mm) (15.3%), and high number of nodes (≥4 positive lymph nodes) (15.8%).

### Discrimination

**Fig. 3:**
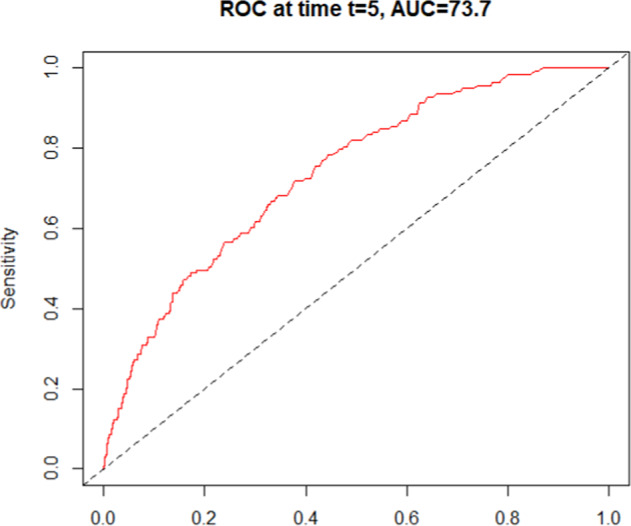
Discriminatory accuracy of PREDICT represented by the area under the receiver-operator characteristic (ROC) curve at the 5-year timepoint in the overall population. ROC receiver-operator characteristic, AUC area under curve.

AUC under the ROC curve was 73.7% (95% CI 69.7–77.8) in the overall population (Fig. 3).

This finding of suboptimal discriminatory accuracy was consistent across all subgroups, ranging from 61.7% (in patients with ≥4 positive lymph nodes) to 77.7% (in patients receiving trastuzumab alone as anti-HER2 therapy) (Table 3). The lowest discriminatory accuracy was observed for patients with high number of nodes (≥4 and 1–3 positive lymph nodes) (Supplementary Figs 1 and 2), and for patients receiving a non-anthracycline-based chemotherapy (61.7%, 64.8%, and 65.2%, respectively). The highest discriminatory accuracy was observed for patients with negative lymph nodes (Supplementary Fig. 3) and for patients receiving trastuzumab alone as anti-HER2 therapy (77.3% and 77.7%, respectively).

**Table 3:** Discriminatory accuracy of PREDICT in the overall population and in subgroups.

		AUC for time-point 5 years (95% CI)
All patients	2794	73.75 (69.73–77.76)
*Type of anti-HER2 therapy*		
Lapatinib + trastuzumab	925	72.37 (64.31–80.42)
Trastuzumab alone	936	77.67 (72.02–83.32)
Trastuzumab followed by lapatinib	933	70.64 (63.51–77.78)
*Type of chemotherapy*		
Non-anthracycline based	322	65.18 (50.36–80.00)
Anthracycline based	2472	74.44 (70.32–78.57)
*Age at randomization*		
≤40	495	76.09 (66.20–85.97)
41–64	1989	73.69 (68.75–78.62)
≥65	310	67.42 (56.95–77.89)
*HR status*		
Negative	1185	71.87 (65.79–77.96)
Positive	1609	76.81 (71.58–82.04)
*Number of positive lymph nodes*		
0	567	77.25 (65.5–89.01)
1–3	945	64.76 (54.58–74.96)
≥4	709	61.74 (55.05–68.43)
*Tumor size (mm)*		
≤20 mm	1248	70.63 (61.83–79.44)
21–50 mm	1356	68.61 (63.27–73.94)
>50 mm	190	72.97 (63.09–82.84)

*AUC* area under the curve, *CI* confidence interval, *CT* chemotherapy, *HR* hormone receptors.

## Discussion

To the best of our knowledge, PREDICT is the only publicly available, free, online tool developed to predict individual prognosis in the specific population of patients with HER2-positive early breast cancer based on traditional and easily retrieved clinical-pathological factors including HER2. In our ALTTO analysis, PREDICT highly underestimated patients’ OS; this finding was consistent across all patient subgroups, with highest absolute differences for patients with hormone receptor-negative disease, nodal involvement, and large tumor size. In terms of discrimination, the accuracy of PREDICT was overall low, with the lowest discriminatory accuracy observed in patients with nodal involvement (≥4 and 1–3 positive lymph nodes), and in patients receiving non-anthracycline-based chemotherapy.

The low performance of this tool raises several questions about the reliability of PREDICT to give prognostic estimation in HER2-positive early breast cancer patients. To potentially explain the reasons for the underestimation of patients’ OS, we can speculate whether the population used to validate this prognostic tool accurately mirrors the real-world population of patients with HER2-positive disease treated in the modern era with effective chemotherapy and anti-HER2 targeted therapies. The prognostic effect of HER2 status was evaluated and incorporated in the PREDICT tool for the first time in October 2011, based on data from the Breast Cancer Association Consortium (BCAC)^6^ consisting in 10,179 cases not exposed to anti-HER2 treatment (Supplementary Table 1). The subsequently developed model (called PREDICT Plus) was then validated in the original British Columbia dataset, a cohort including 203 HER2-positive breast cancer patients^7^. In this latter cohort, PREDICT demonstrated an improved ability to estimate breast cancer-specific and overall survival in HER2-positive patients, compared to other prognostication tools such as PREDICT and Adjuvant! Online^7^. In the HER2-positive cohort of the British Columbia dataset, the observed 10-year OS was 44.3%, and none of the included patients had received trastuzumab^7^. A further step forward, was the inclusion in PREDICT of the estimates of benefit from adjuvant trastuzumab, with its proportional reduction of 31% in the mortality rate up to five years. These estimates were based on the results of four clinical trials: FinHER^10^, HERA^11^, B31/N9831^12,13^, and BCIRG006^14^ (Supplementary Table 2).

Patients with HER2-positive early breast cancer are experiencing a consistent shift towards better survival across the years, mainly due to the increasingly effective local and systemic therapies available in this setting. This change might not be reflected by a prognostic tool developed and validated 10 years ago. In particular, newer drugs like pertuzumab and T-DM1 have become available for many patients developing disease progression after treatment in the ALTTO trial. These two drugs improve OS in metastatic patients and may contribute to the “better-than-predicted” OS^15,16^. Moreover, the current standard of care for early breast cancer is even superior to the treatment received by many patients in the ALTTO study, including neoadjuvant therapy with pertuzumab, adjustment of adjuvant therapy based on pathological response to neoadjuvant therapy (i.e., T-DM1 for patients who do not achieve pathological complete response) and considering extended adjuvant anti-HER2 therapy with neratinib and endocrine therapy for patients with hormone receptor-positive disease. As such, the discordance between OS estimated by PREDICT and the current real-world OS is expected to be even higher. Therefore, our results suggest that the current version of PREDICT should be used with caution for prognostication in HER2-positive early breast cancer patients treated in the modern era with effective chemotherapy and anti-HER2 targeted therapies.

It should be also considered that at least part of the discordance observed between the observed and predicted 5-yr OS by PREDICT could be due to the differences existing between a highly selected population enrolled in a clinical trial and the real-world patient population, which might have slightly different prognosis^17,18^. Clinical trials have a strong internal validity, but their external validity could be weaker, particularly in the case of narrow inclusion criteria. For this reason, findings from clinical trials might overestimate outcomes as compared to real-world practice. Due to differences in the distribution of age, comorbidity status, and overall health, differences between predicted and observed OS in a clinical trial sample as compared to real-world data are expected. Consistently with our findings, an independent validation of PREDICT on data from real-world patients led by Gray and colleagues showed a general pattern of overestimation of mortality (expected and observed 5-year mortality: 15.3% and 14.5%, respectively), although not focusing specifically on HER2-positive disease^19^.

Additionally, prognostication estimates of PREDICT are provided as OS rates. Although OS is an important endpoint, being free from any ambiguity in its definition, it could be influenced by several variables (competing risks) not strictly related to breast cancer and not considered in PREDICT, such as comorbidities and performance status^20^. Non-cancer deaths may not entirely reflect tumor biology, aggressiveness, and responsiveness to therapy^20^. On the other hand, the more aggressive the disease, the higher the relevance of OS. Indeed, HER2-positive breast cancer tend to develop more early recurrences compared to hormone receptor positive/HER2-negative disease, thus having an undoubtedly more relevant impact on OS^21^.

In our analysis, the highest absolute differences between observed and predicted OS were observed for patients with hormone-receptor negative disease, larger tumor size, and high number of nodes (≥4 positive lymph nodes), namely those patients traditionally considered at higher risk of relapse. Further investigations are urgently needed to better predict prognosis of these patients. Of note, despite the traditional stigma of poor prognosis for patients with high-risk HER2-positive breast cancer, recent clinical trials have shown good outcomes also for this high-risk subset of patients^22^.

The prediction of prognosis in patients with early breast cancer is an issue of paramount importance, not only in hormone receptor-positive/HER2-negative disease, where prognostication may settle whether adjuvant chemotherapy should be administered or not, but also in HER2-positive disease. Indeed, although in HER2-positive breast cancer almost all patients deserve chemotherapy as per standard of care, a reliable prognostic estimation has several implications, from the planning of premenopausal patients’ reproductive life (e.g. affecting the choice of having or not a pregnancy later on^23^), to a therapeutic perspective (adoption of escalation or de-escalation treatment strategies, including type of chemotherapy to be administered together with anti-HER2 treatment and use of extended adjuvant endocrine therapy in hormone receptor-positive disease^24^).

Several molecular assays are now available for hormone receptor-positive/HER2-negative breast cancer^25^, and, recently, some molecular assays have been also developed for HER2-positive disease^26^.

It is likely that these assays will refine prognostication beyond what can be provided by clinical prognostic models like PREDICT^27,28^, and their increasing use, as a consequence, will reduce reliance on tools like PREDICT. Nevertheless, one strength of PREDICT is the fact that it is “free” and easy to use in everyday clinical practice, and its integration with molecular assay could provide a more complete prognostic evaluation of each single patient. Recently, Prat et al. developed a new prognostic score, HER2DX, based on the combination of clinical-pathological and molecular characteristics of the tumor (nodal and tumor stage, the number of stromal tumor-infiltrating lymphocytes, PAM50 subtypes, and expression of 13 genes relating to proliferation and underlying subtype-related biology)^26,29^. This was the first attempt to build a combined prognostic score based on clinicopathological and genomic variables in early-stage HER2-positive breast cancer, using tumor samples from the phase 3 Short-HER trial^30^. However, the HER2DX prognostic model is still immature to be used as biomarker, and future clinical validations are warranted in order to establish its use in different scenarios, especially in the neoadjuvant setting.

Our study has some limitations that should be acknowledged. First, this is an unplanned exploratory analysis. Second, some information (including prognostic factors like the proliferation index Ki67 and type of method for breast cancer detection) were not available in the ALTTO database and could not be included in the model. Third, PREDICT did not allow for estimates of dual-targeted anti-HER2 therapy efficacy, and, in particular, does not provide estimates for lapatinib use. However, our subgroup analysis confirmed that PREDICT still underperforms for patients treated with trastuzumab alone. Additionally, PREDICT tool does not consider the presence of comorbidities and/or the patient performance status, thus further limiting the possibility to compare predicted vs. observed outcomes using a clinical trial sample. Finally, only the point estimates by PREDICT, without its range, were included in the present analysis.

On the other hand, our study has several strengths. Our results derive from a large cohort (*n* = 2794) of patients enrolled in the largest, randomized adjuvant trial ever conducted in the field of HER2-positive breast cancer. We included only patients receiving adjuvant trastuzumab-based therapy started concurrently with modern chemotherapy. Trial sample size allowed the exploration of relevant patient subgroups. All data used for the analyses were prospectively collected during the trial conduction, as detailed in the study protocol.

In conclusion, in patients with HER2-positive early breast cancer enrolled in the ALTTO trial and treated with modern chemotherapy and trastuzumab-based therapies, the PREDICT score highly underestimated OS. The suboptimal performance of this prognostic tool was observed irrespective of type of anti-HER2 treatment, type of chemotherapy regimen, age of the patients at the time of diagnosis, central hormone receptor status, pathological nodal status, and pathological tumor size. Our results suggest that the current version of PREDICT should be used with caution to give prognostic estimation in HER2-positive early breast cancer patients treated in the modern era with effective chemotherapy and anti-HER2 targeted therapies. The further improvement of therapeutic strategies expected in the next future will likely increase the survival of patients with HER2-positive early breast cancer, thus requiring the current version of PREDICT to be updated to provide reliable prognostic estimation in these patients.

## Methods

### Study design and patients

Details of the ALTTO trial study design were previously published^31^. Shortly, the ALTTO trial (Breast International Group [BIG] 2-06/EGF106708 and North Central Cancer Treatment Group [Alliance] N063D) was an international, open-label, randomized phase III study testing the use of trastuzumab and/or lapatinib as adjuvant anti-HER2 therapy in patients with HER2-positive early breast cancer.

Primary tumor samples from all patients were centrally tested to assess HER2^32^ and hormone receptor status^33^.

Eligible patients were randomized to one of four anti-HER2 treatment arms: trastuzumab alone, lapatinib alone, sequential treatment with trastuzumab for 12 weeks followed by a 6-week washout period before other 34 weeks of lapatinib, and dual anti-HER2 blockade with trastuzumab plus lapatinib. The CONSORT diagram of the ALTTO study is reported in the ALTTO primary analysis paper.

Anti-HER2 treatment could be administered as per physician’s choice following chemotherapy completion (design 1), or concomitantly, either with a taxane after anthracycline-based chemotherapy (design 2) or with 6 cycles of docetaxel and carboplatin in an anthracycline-free regimen (design 2B). In all treatment arms, adjuvant anti-HER2 therapy was administered for 1 year.

In 2011, after the first interim analysis, the lapatinib arm was closed and patients were offered adjuvant commercial trastuzumab^31^.

In the present analysis, in order to reflect current clinical practice in this setting, only patients who received concurrent chemotherapy (design 2 and design 2B) and who received trastuzumab-based anti-HER2 therapy (i.e. trastuzumab alone arm, trastuzumab followed by lapatinib arm and trastuzumab plus lapatinib arm) were included. All patients originally assigned to the lapatinib alone arm, and those who received anti-HER2 therapies at the completion of all chemotherapy (sequential treatment, design 1) were excluded.

### Ethics section

All patients signed a written informed consent prior to enrollment in ALTTO. The project proposal of the present exploratory analysis was submitted and approved by the ALTTO Steering Committee.

### Study objectives

The primary objective of the current analysis was to investigate the prognostic performance of PREDICT in breast cancer patients with early-stage HER2-positive disease treated with modern chemotherapy and concurrent trastuzumab-based anti-HER2 therapy.

Secondary objectives were to investigate the prognostic performance of PREDICT according to the type of anti-HER2 treatment received (trastuzumab alone, trastuzumab followed by lapatinib and, trastuzumab plus lapatinib), type of chemotherapy regimen received (anthracycline-based chemotherapy regimens vs. non-anthracycline-based chemotherapy regimens), age of patients at the time of diagnosis (age ≤ 40 years vs. age 41–64 vs. age ≥65 years), central hormone receptor status (hormone receptor -positive vs. negative), pathological nodal status (node-negative vs. node-positive disease [1–3 positive nodes] vs. node-positive disease [≥4 positive nodes]), and pathological tumor size (small [≤2 cm] vs. medium [2–5 cm] vs. large [>5 cm] disease).

### Data extraction

PREDICT estimates for each patient were calculated by one investigator blinded to patient outcomes. Patient and tumor characteristics, as well as administered adjuvant anticancer treatments, were entered in the PREDICT v.2.2 program to calculate the predicted 5-year OS for each patient. Detection modality and Ki67 status were considered “unknown” for all patients (as these variables were not collected as part of the ALTTO trial).

The most updated ALTTO database was used for this analysis^9^, which corresponds to at least 5-year follow-up for every single patient.

### Statistical analysis

The present analysis should be considered as exploratory, since it was not preplanned in the study protocol and the power of the statistical analyses performed was not pre-specified.

The prognostic performance of PREDICT was evaluated by assessing the following endpoints: i) calibration, defined as the agreement between the predicted and observed survival rates, and ii) discriminatory accuracy, defined as the ability of distinguishing individuals who will survive 5 years compared to those who will not (i.e. the ability to discern patients with good outcomes from those with poor outcomes at the individual patient level).

The observation time for each patient was defined as the time between the date of diagnosis and an event. OS event was defined as death from any cause.

The median predicted 5-year OS was calculated from individual predicted outcomes by PREDICT v. 2.2.

For assessing calibration, the median predicted 5-year survival probabilities (by PREDICT) were compared with the observed 5-year survival rates (as obtained by Kaplan-Meier curves). We had to use the median 5-year prediction instead of the mean 5-year prediction, due to the skewness in the distribution, i.e. mean 5-year prediction was 83.6% while median 5-year prediction was 88.0%, and thus the mean predicted 5-year survival probability underestimated the center of the distribution. Therefore, we used the median as a robust estimator of the center of the distribution. Using the standard error as obtained by the Kaplan-Meier curve, we calculated 95% CI for the difference in predicted vs. observed 5-year survival. Calibration plots for PREDICT were constructed by visualizing mean predicted vs. observed survival outcomes by deciles of predicted outcomes.

For assessing discriminatory accuracy, the area under the receiver-operator characteristic curve (AUC under the ROC) and corresponding 95% CI for 5-year predicted OS were calculated. The AUC translates into the probability that the predicted outcome of a randomly selected patient who indeed had that outcome is higher than that of a patient who did not; the higher the AUC, the better the tool is at identifying patients with a better survival.

Subgroup analyses were performed to investigate the prognostic performance of PREDICT according to the type of anti-HER2 treatment and chemotherapy received, age at the time of diagnosis, central hormone receptor status, pathological nodal status, and pathological tumor size.

Statistical analysis was performed by L.A. using SAS 9.4 statistical software (SAS Institute, Cary, NC) and R.

### Reporting summary

Further information on research design is available in the Nature Research Reporting Summary linked to this article.

## Supplementary information

Supplementary material

Reporting Summary

## Data Availability

Data can be made available upon reasonable request. Data and results from the Data Centre at Institut Jules Bordet in Brussels (Belgium) can be made available upon approval of a research proposal.
