# Supplementary material for: PREDICT underestimates survival of patients with HER2-positive early-stage breast cancer
Source: NPJ Breast Cancer. 2022 Jul 20;8:87. doi: 10.1038/s41523-022-00452-8 (PMC9300724; doi:10.1038/s41523-022-00452-8)
Supplement: Supplementary file 1 — Supplementary material [file 41523_2022_452_MOESM1_ESM.pdf]

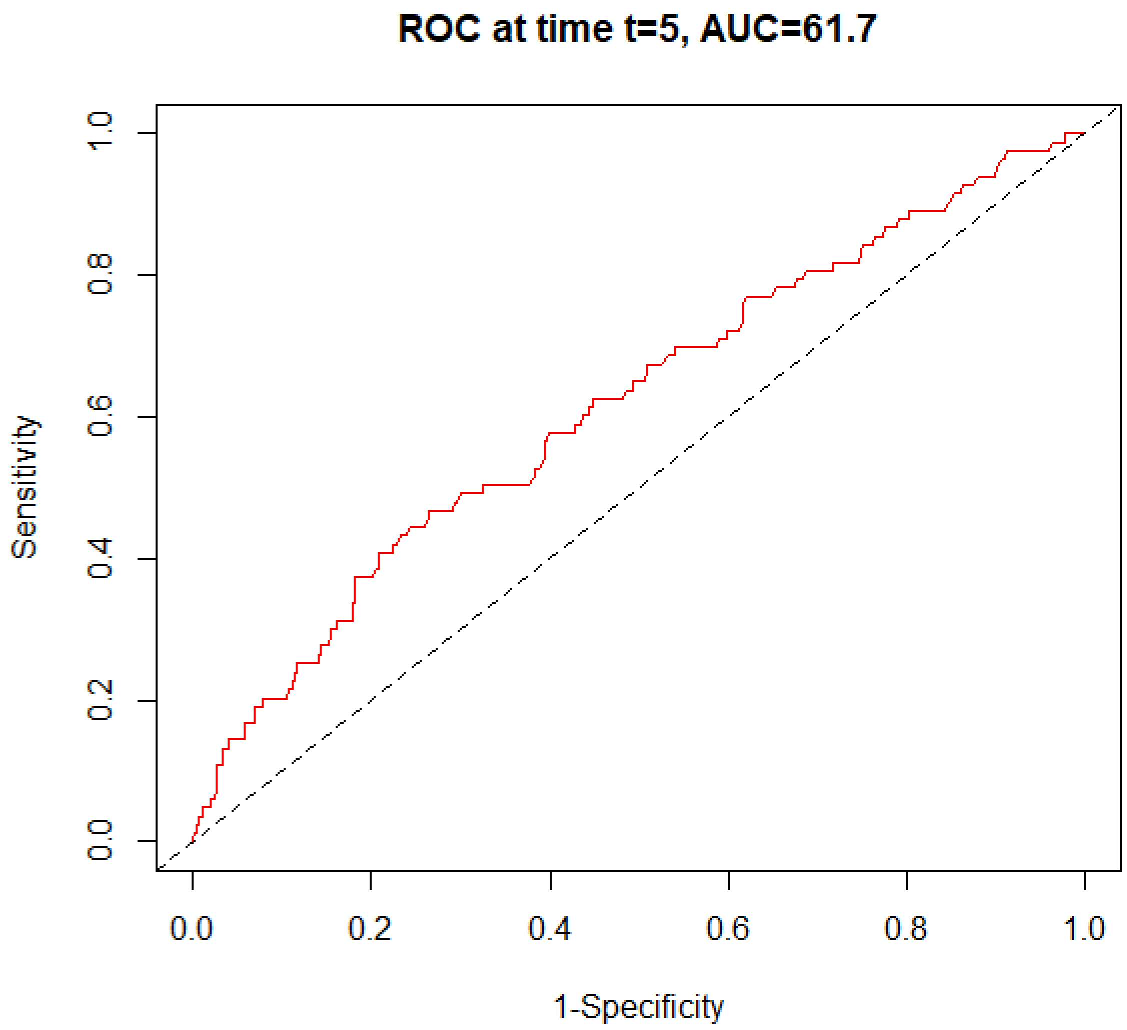

Supplementary Figure 1. Discriminatory accuracy of PREDICT+ in patients with ≥4 positive lymph nodes.
Abbreviations: ROC: receiver-operator characteristic; AUC: area under curve.

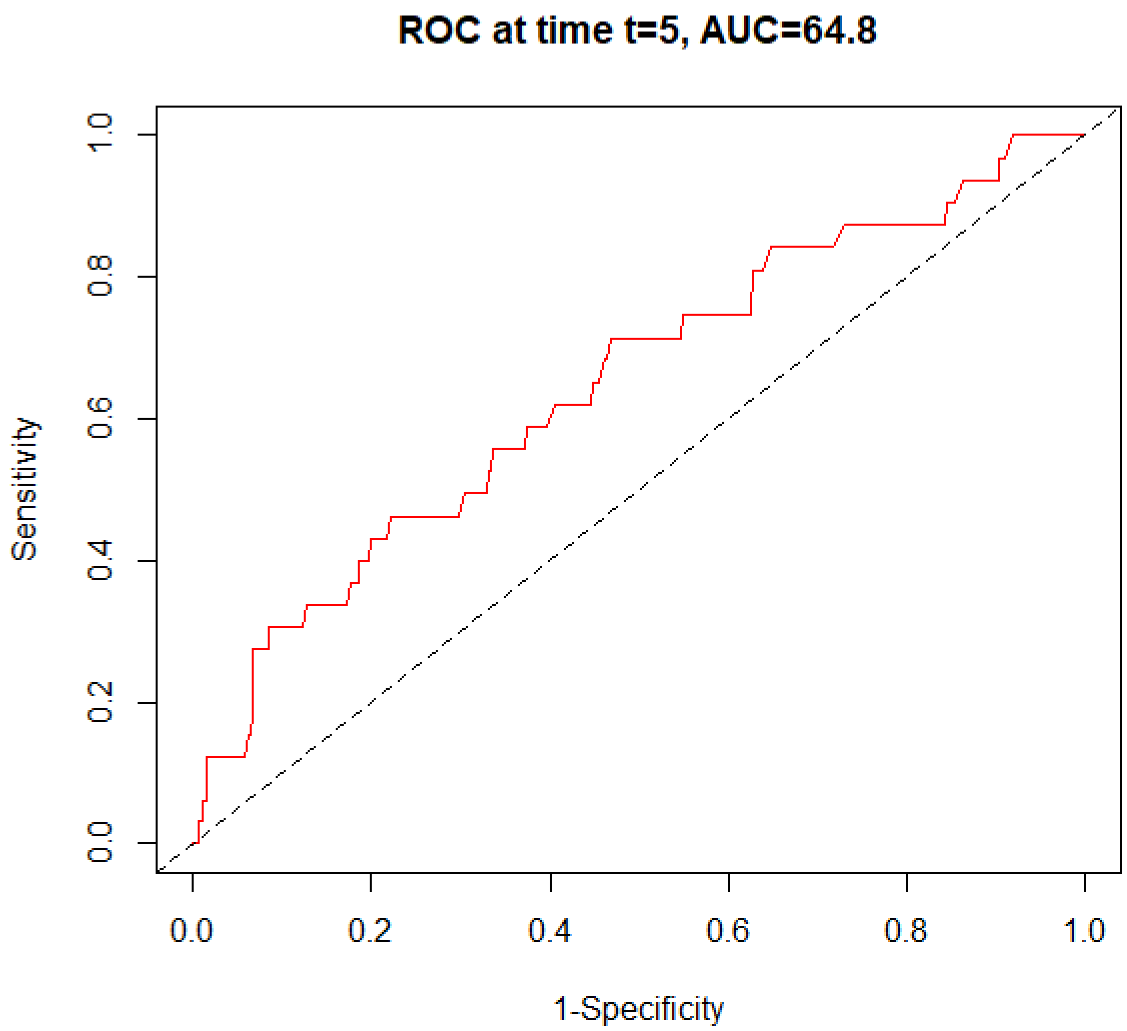

Supplementary Figure 2. Discriminatory accuracy of PREDICT+ in patients with 1-3 positive lymph nodes.
Abbreviations: ROC: receiver-operator characteristic; AUC: area under curve.

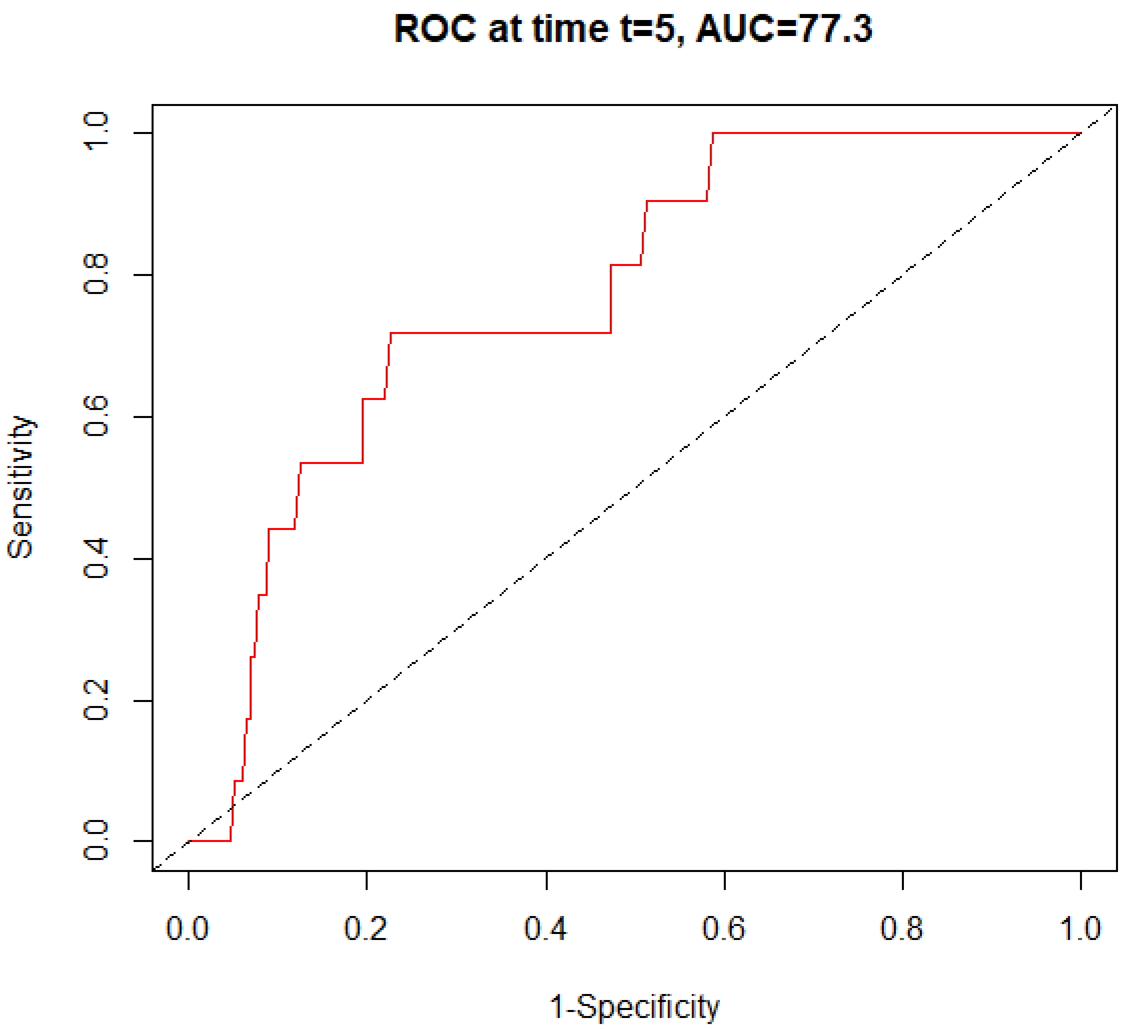

Supplementary Figure 3. Discriminatory accuracy of PREDICT+ in patients with negative lymph nodes.
Abbreviations: ROC: receiver-operator characteristic; AUC: area under curve.

Supplementary Table 1. Studies and cohorts involving patients with HER2-positive breast cancer used for the development and validation of PREDICT+.

**Study/Cohort**	**Population characteristics**	**Aim**
Breast Cancer Association Consortium (BCAC), 2011	N= 10,179, none treated with trastuzumab	Estimates for the prognostic effect of HER2 status
British Columbia dataset, Canada cohort (BJC, 2012)	N=1,653 (HER2-positive n=203), none treated with trastuzumab	Validation
FinHER (Joensuu et al, 2009)<br>HERA (Smith et al, 2007)<br>B31/N9831 (Romond et al, 2005; Perez et al, 2010)<br>BCIRG006 (Slamon et al, 2011)	Early HER2-positive breast cancer (details for each study are reported in Table 5)	Evaluation and incorporation of benefit from trastuzumab

Supplementary Table 2. Studies providing estimates of trastuzumab benefit for PREDICT+ and comparison with the present study.

Study	Population	Treatment	Outcome
**FinHER** (Joensuu et al, 2009)	node-positive or high-risk node-negative<br><br>N=1010 (overall)<br>N=232 (HER2+)	HER2+ cohort:<br>Arm A: docetaxel(x3) +/- trastuzumab →FEC(x3)<br>Arm B: vinorelbine(x3) +/- trastuzumab →FEC(x3)	HER2+ cohort:<br>5-yr OS: 91.3% (CT+trastuzumab) vs 82.3% (CT, no trastuzumab)<br><br>5-yr OS: 94.4% in trastuzumab, docetaxel, FEC cohort vs 82.0% in docetaxel, FEC, no trastuzumab
**HERA** (Smith et al, 2007)	HER2+ node positive or high-risk node negative breast cancer N=3401	Standard (neo)adjuvant CT* +/- trastuzumab for 1 year	3-yr OS: 92.4% (trastuzumab) vs 89.7% (no trastuzumab)
**B31/N9831** (Romond et al, 2005; Perez et al, 2010)	surgically removed HER2+ BC N=3951	AC→paclitaxel +/- trastuzumab	4-yr OS: 85.6% in control group and 93.0% in trastuzumab group
**BCIRG006** (Slamon et al, 2011)	HER2+ early BC N=3222	AC→docetaxel +/- trastuzumab or docetaxel and carboplatin + trastuzumab	5-yr OS: 87% (AC→docetaxel) vs 92% (AC→docetaxel + trastuzumab) vs 91% (docetaxel and carboplatin + trastuzumab)
**ALTTO PREDICT+ Study**	HER2+ early BC N=2794	Design 2: Anthracycline→ taxane + anti-HER2 (trastuzumab-based)<br>Design 2B: docetaxel+carboplatin + anti-HER2 (trastuzumab-based)	5-yr observed OS: 95.0%<br>5-yr predicted OS by PREDICT+: 88.0%

*in 68% of pts anthracycline-based, no taxanes, in 26% anthracyclines+taxanes, 6% no anthracyclines

Abbreviations: HER2+: HER2-positive, FEC: 5-fluorouracil, epirubicin and cyclophosphamide, OS: overall survival, CT: chemotherapy, BC: breast cancer, AC: doxorubicin and cyclophosphamide.
